# Signaling pathways involved in colorectal cancer progression

**DOI:** 10.1186/s13578-019-0361-4

**Published:** 2019-12-02

**Authors:** Zahra Koveitypour, Farnoush Panahi, Mehrdad Vakilian, Maryam Peymani, Farzad Seyed Forootan, Mohammad Hossein Nasr Esfahani, Kamran Ghaedi

**Affiliations:** 1 Department of Modern Biology, ACECR Institute of Higher Education (Isfahan Branch), Isfahan, Iran; 2 Department of Biology, Faculty of Basic Sciences, Shahrekord Branch, Islamic Azad University, P.O. Box: 88137-33395, Shahrekord, Iran; 3 Department of Cell and Molecular Biology and Microbiology, Faculty of Biological Science and Technology, University of Isfahan, Isfahan, Iran; 4 Department of Cellular Biotechnology, Cell Science Research Center, Royan Institute for Biotechnology, ACECR, P.O. Box: 816513-1378, Isfahan, Iran; 5 Legal Medicine Research Center, Legal Medicine Organization, Tehran, Iran; 6 Department of Cell Regeneration and Advanced Therapies, Andalusian Center for Molecular Biology and Regenerative Medicine (CABIMER), University of Pablo de Olavide-University of Seville-CSIC, Seville, Spain; 7 Department of Cellular Biology, Genetics and Physiology, Faculty of Science, University of Malaga (UMA), Malaga, Spain

**Keywords:** Colorectal cancer, EGFR, MAPK, Notch, TGF-β

## Abstract

Colorectal cancer (CRC) is the fourth leading cause of the worldwide cancer mortality. Different molecular mechanisms have been attributed to the development and progress of CRC. In this review, we will focus on the mitogen-activated protein kinase (MAPK) cascades downstream of the epidermal growth factor receptor (EGFR), Notch, PI3K/AKT pathway, transforming growth factor-β (TGF-β), and Wnt signaling pathways. Various mutations in the components of these signaling pathways have been linked to the development of CRC. Accordingly, numerous efforts have been carried out to target the signaling pathways to develop novel therapeutic approaches. Herein, we review the signaling pathways involved in the incidence and progression of CRC, and the strategies for the therapy targeting components of signaling pathways in CRC.

## Introduction

Colorectal cancer is the second most common type of malignancy and the fourth leading cause of the cancer-related death worldwide [1]. In terms of cancer etiology, and CRC as a particular example, the mechanism of cancer development is a complex multistage process, involving sequential mutational events occurring along with progression of the cancer [2]. As mentioned, EGFR/MAPK, Notch, PI3K, TGF-β and Wnt signaling pathways are implicated in the regulation of several biological processes, including cell proliferation, differentiation, angiogenesis, apoptosis and survival [3].

Various genes, are associated to the signaling pathways, which have been frequently reported to be dysregulated due to mutations or the altered function of their products in the CRC. Several studies have pinpointed those genes responsible for increased proliferation, invasion, progression or inhibition of apoptosis in CRC cells, including *EGFR*, *RAS*, *RAF*, *Notch*-*1*, *Jagged*-*1*, *PIK3CA*, *PTEN*, *TGFBR2*, *TGFBR1*, *SMADs*, *AXIN* and *CTNNB1* [4–7]. Recent progress in deciphering the crosstalk between signaling pathways in CRC is remarkable. Therefore, perceiving the connections between these pathways may trigger development of novel therapeutic or preventive approaches against CRC.

## EGFR/MAPK signaling pathway in CRC

**Fig. 1:**
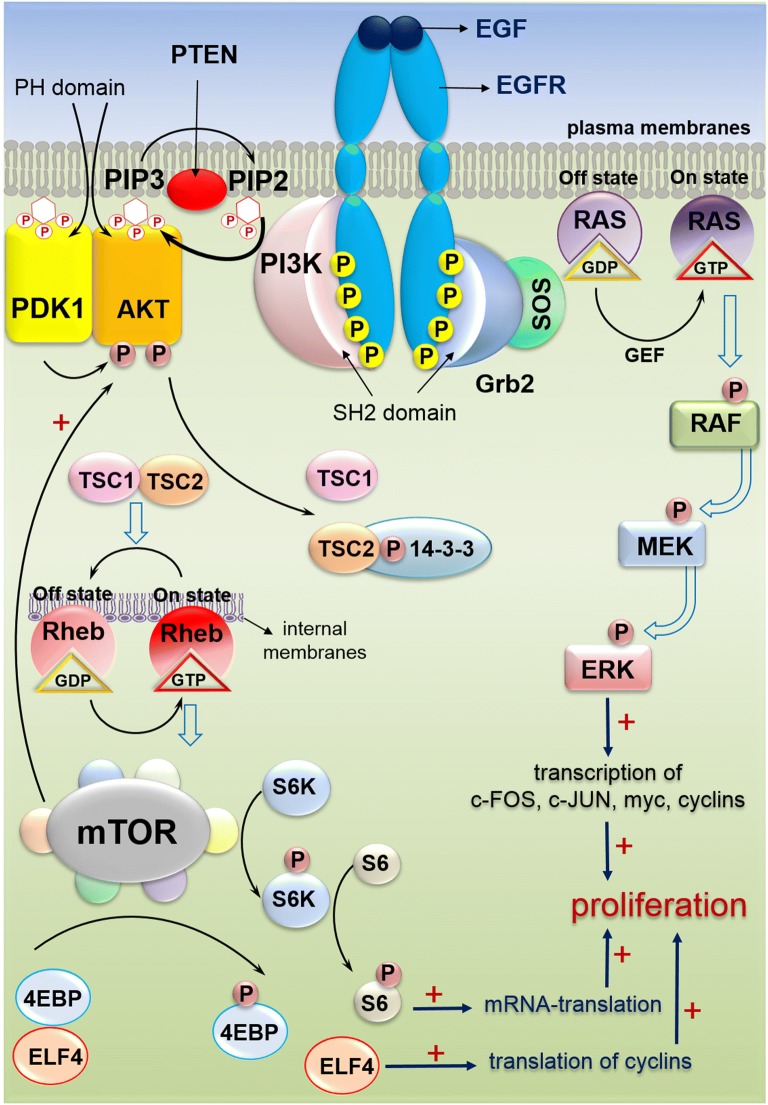
EGFR and PI3K signaling pathways in CRC. The binding of EGF to the extracellular domain of EGFR, induces dimerization, and activation of intrinsic kinase activity. The proteins, those are recruited to active EGFR include a number of Src homology 2 (SH2) proteins. One of the adaptor proteins, GRB2 recruits SOS to the membrane. SOS activates GDP/GTP exchange which recruits RAF to the membrane. RAF phosphorylates MEKs, which then activates the extracellular signal regulated kinase (ERK). Phosphorylated ERK translocates to nucleus and activates transcription factors leading to expression of the target genes such as c-FOS, c-JUN and myc [4]. GRB2 recruits PI3Ks, another major mediator of EGFR signaling pathway. PI3Ks converts PIP2 to PIP3. PIP3 binds to PH domain of AKT and recruits it to plasma membrane. PDK1 phosphorylates AKT which in turn regulates the activity of various proteins that mediate cell survival. Activated AKT inhibits TSC2 via phosphorylation. Inactive TSC1/2 is unable to bind RAS homolog enriched in brain (RHEB), which subsequently enables its activation of mTORC1 at the surface of lysosome. Upon activation, mTORC1 regulates many cellular functions, such as cell growth, protein synthesis and autophagy via S6 kinase (S6K; RPS6K) and eukaryotic translation initiation factor 4E-binding protein 1 (4E-BP1; EIF4EBP1) [68]

EGFR is a transmembrane protein belonging to the ErbB family of receptors, which functions as a receptor tyrosine kinase (RTK). ErbB family of receptors has four closely related members, including ErbB-1 (HER1/EGFR), ErbB-2 (HER2), ErbB-3 (HER3), and ErbB-4 (HER4) [8]. The EGFR signaling pathway participates in many cellular processes, including the growth, proliferation and survival of normal cells. Disruption of EGFR pathway modulates growth, proliferation, survival and metastasis of neoplastic cells [9]. MAPK is a member of the large family of Ser/Thr kinases, which triggers multiple rounds of hierarchical phosphorylation-activating kinase circles, from the cell surface to the nucleus. Three major subfamilies of MAPK are the extracellular-signal-regulated kinases (ERK MAPK, Ras/Raf1/MEK/ERK), the c-Jun N-terminal or stress-activated protein kinases (JNK or SAPK), and MAPK14 [10]. In this article, the ERK MAPK pathway will be reviewed. Many growth-factor receptors, including EGFR, are located upstream of MAPK pathways [11]. There are three Ras small GTPases including H-Ras, N-Ras, and K-Ras [12]. Also, A-Raf, B-Raf and C-Raf (Raf1) are several types of Raf [13]. Following ligand binding, the homo or heterodimer types of receptors appear, which contribute to induction of auto-phosphorylation of selected tyrosine residues on receptor [14]. The EGFR signaling cascade has an adaptor protein complex containing the growth factor receptor bound protein 2 (Grb2) and the son of seven-less (SOS). This complex activates Ras-GTP by binding to phosphorylated tyrosine residues. After RAS activating, there is a cascade of activating RAF MEK and ERK through phosphorylation (Fig. 1). It has been suggested that the Ras–Raf–ERK signaling pathway contributes to the control of cell growth, differentiation, and survival. When this pathway is dysregulated, it can lead to malignant transformation and tumor progression through the increased cell proliferation, prolonged survival, angiogenesis, anti-apoptosis, invasion, and metastasis. As noted, EGFR/MAPK signaling pathway has been related to the oncogenic processes and therefore plays important role in tumor growth and the progression of CRC [3, 4]. Aberrant expression of this pathway has been reported as target for CRC treatment [14, 15].

## Notch signaling pathway in CRC

**Fig. 2:**
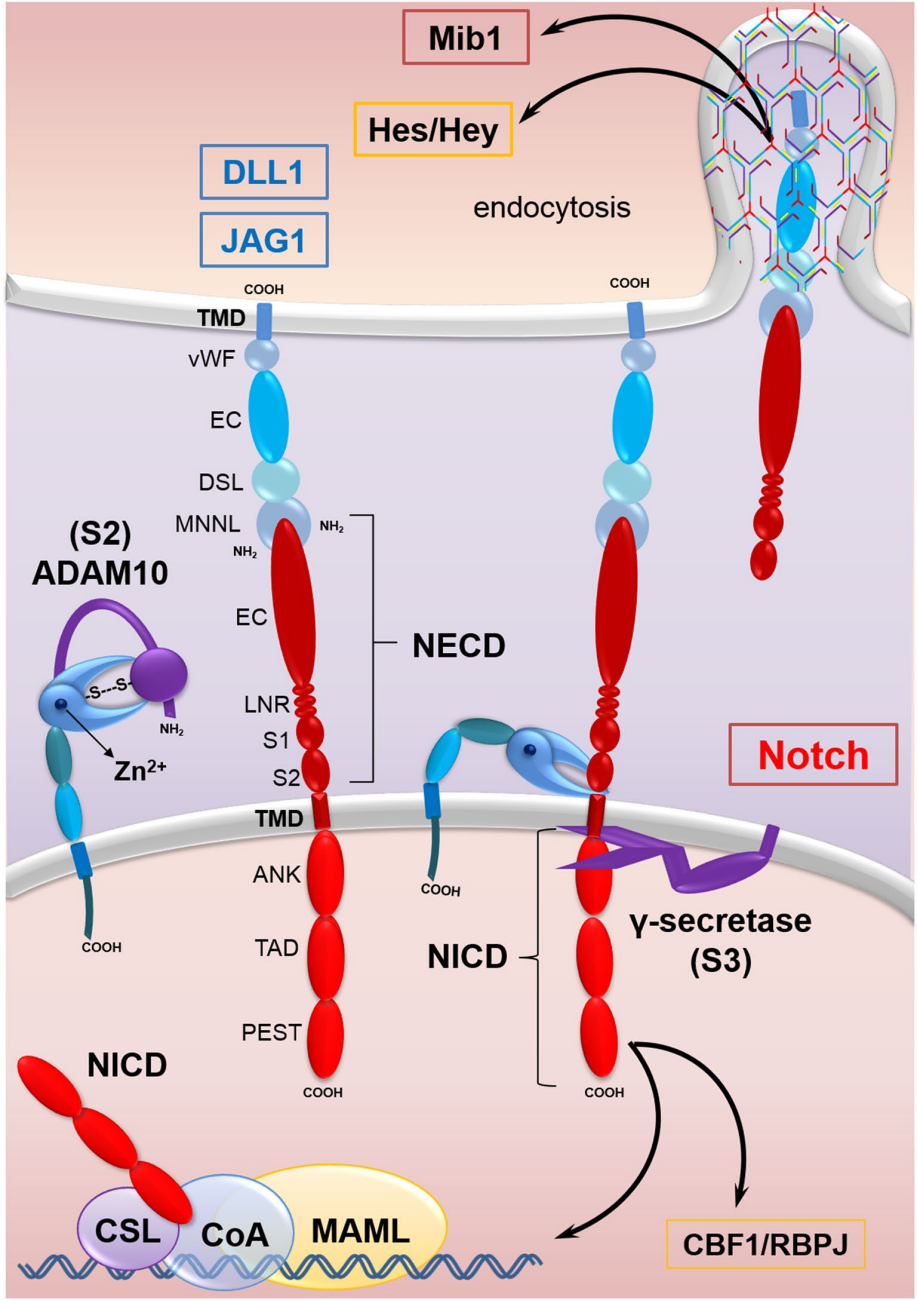
Notch signaling pathways in CRC. Mind bomb-1 (Mib1), an E3 ubiquitin ligase, promotes the endocytosis of Notch ligands. The extracellular domain of Notch ligands (DLL1 or JAG1 shown) consists of an N-terminal MNNL domain, DSL domain, EC and vWF [69]. The Notch receptors are transmembrane proteins containing extracellular or NECD (EC, LNR, S1 and S2), transmembrane domain or TMD and intracellular domains or NICD (ANK, TAD and PEST) [19]. Activation of Notch pathway is initiated by binding of Notch ligands to Notch receptors which leads to their conformational changes. Then, S2 cleavage site is exposed for ADAM to remove the extracellular region. Subsequently, S3 cleavage occurs by γ-secretase which removes the transmembrane region and releases NICD. Then NICD translocates into the nucleus, and binds to the inactive CSL transcription factor which forms a complex [20]. Instead co-activators, such as MAML could bind to CSL, resulting in activating of this transcriptional complex [21]. Finally, expression of target genes, including Hes family, is induced [18]

Notch pathway is one of highly conserved cellular pathways responsible for direct cell to cell interaction. Proper function of Notch pathway is essential for normal cell development, differentiation, proliferation and apoptosis [16]. Notch signaling pathway consists of at least five ligands including *Jagged*-*1*, *Jagged*-*2*, *Delta*-*like*-*1* (*Dll*-*1*), *Delta*-*like*-*3* (*Dll*-*3*), and *Delta*-*Like*-*4* (*Dll*-*4*), as well as four receptors: *Notch*-1, -2, -3 and -4. Notch signaling pathway, also contains several downstream target genes including *p21, Hes*-*1*, *Deltex* [17, 18]. The Notch ligands are the single-pass transmembrane proteins of DSL family that contain EGF-like repeats. Notch receptors are transmembrane proteins containing both types of extracellular and intracellular domains [19]. When Notch ligands bind to the Notch receptors of target cell, Notch signaling activation will be started, through activation of γ-secretase protein complex and cleaving the Notch receptors. This step is essential for the production of the active form of Notch, Notch intracellular domain (NICD) [18]. Then, NICD translocates into the nucleus, and binds to the inactive CSL (CBF-1/Suppressor of the hairless/LAG1) transcription factor which forms a complex [20]. The co-repressors previously bound to the CSL will be displaced after formation of this complex. Instead co-activators such as mastermind-like proteins (MAML), p300 and the histone acetyltransferase (HAT) will be bound to CSL, resulting in activation of this transcriptional complex [21]. Finally, expression of target genes, including *Hes* family, will be induced (Fig. 2) [18]. Notch signaling mediates the maintenance of intestinal development and homeostasis through the regulation of the differentiation of colonic goblet cells and stem cells/progenitor cells [22]. Previous study has reported that Notch ligands, Notch receptors 1–4, and some downstream targets of Notch signaling (Hes-1, Deltex and NICD) are overexpressed in colorectal cancer cells [23]. Notch signaling pathway has a complicated role in tumorigenesis [7]. It could have either an oncogenic or tumor suppressor role, depending on the context of cancer cells [20]. In most cases, Notch signaling pathway functions as tumor promoter but in a few cases acts as a tumor suppressor. This discrepancy is probably due to Notch-1, which was diagnosed at various tumor stages. In addition, it has been reported that Notch-1 plays an oncogenic role in CRC cells [7]. A recent study has been reported that Notch expression in the primary stage of CRC is relatively higher than the later stage [20]. Notch signaling promotes CRC through regulating the cell cycle and cell apoptosis by regulation of p21 and PUMA genes [7]. Therefore, one of the therapeutic benefits of CRC may be the inhibition of Notch signaling [17, 20].

## PI3K signaling pathway in CRC

PI3K/Akt is an important intracellular signal pathway responsible for a variety of cellular activities such as cell growth, proliferation, differentiation and migration [24]. PI3K, is one of the pathways, which is activated by EGFR signaling. PI3K is a heterodimeric molecule consisting of three classes, class I–III, which can be distinguished by differences in their structures and functions. Type class Ia is most implicated type in human cancer. This class contains two subunits for PI3K, one regulatory (p85) and one catalytic (p110) subunit. Three genes, *PIK3R1*, *PIK3R2* and *PIK3R3*, are encoding different isoforms of the p85, and different types of p110 including alpha, beta, gamma and delta which produce PIK3CA, PIK3CB and PIK3CD, respectively [25]. Akt, a serine/threonine protein kinase (Ser/Thr kinase), mediates the PI3K effects on tumor growth and progression. Thus, Akt is a downstream effector of PI3K. The phosphorylation of Akt has been associated with the cell proliferation and apoptosis inhibition in human CRCs. The inhibition of PI3K/Akt pathway has been used for cancer treatment. For this aim, many molecules are being developed [26]. The activation of PI3K could also be triggered by stimulating the extracellular factors via receptor tyrosine kinases (RTK) or stimulating the activation of Ras. When p85 binds to phosphotyrosine residues of RTK at intracellular part, its inhibitory effect on P110 will be removed, which consequently causes PI3K activation. Activated PI3K phosphorylates phosphatidylinositol 4, 5 biphosphate (PIP2) and generates phosphatidylinositol 3, 4, 5 triphosphate (PIP3). PIP3 is then activates AKT which results in the cell proliferation and cell survival. AKT regulates downstream targets such as mTOR, which promotes angiogenesis, protein translation, growth and metabolism. Phosphatase and tensin homologue protein (PTEN), a tumor suppressor molecule, downregulates the PI3K pathway through dephosphorylating of PIP3 [25]. Overall, it has been reported that PI3K signaling pathway serves an oncogenic role in the initiation and progression of CRC [27]. There are several reports that targeted inhibition of this pathway causes reduction in CRC cell growth and increase in apoptosis [26].

## TGF-β signaling pathway in CRC

**Fig. 3:**
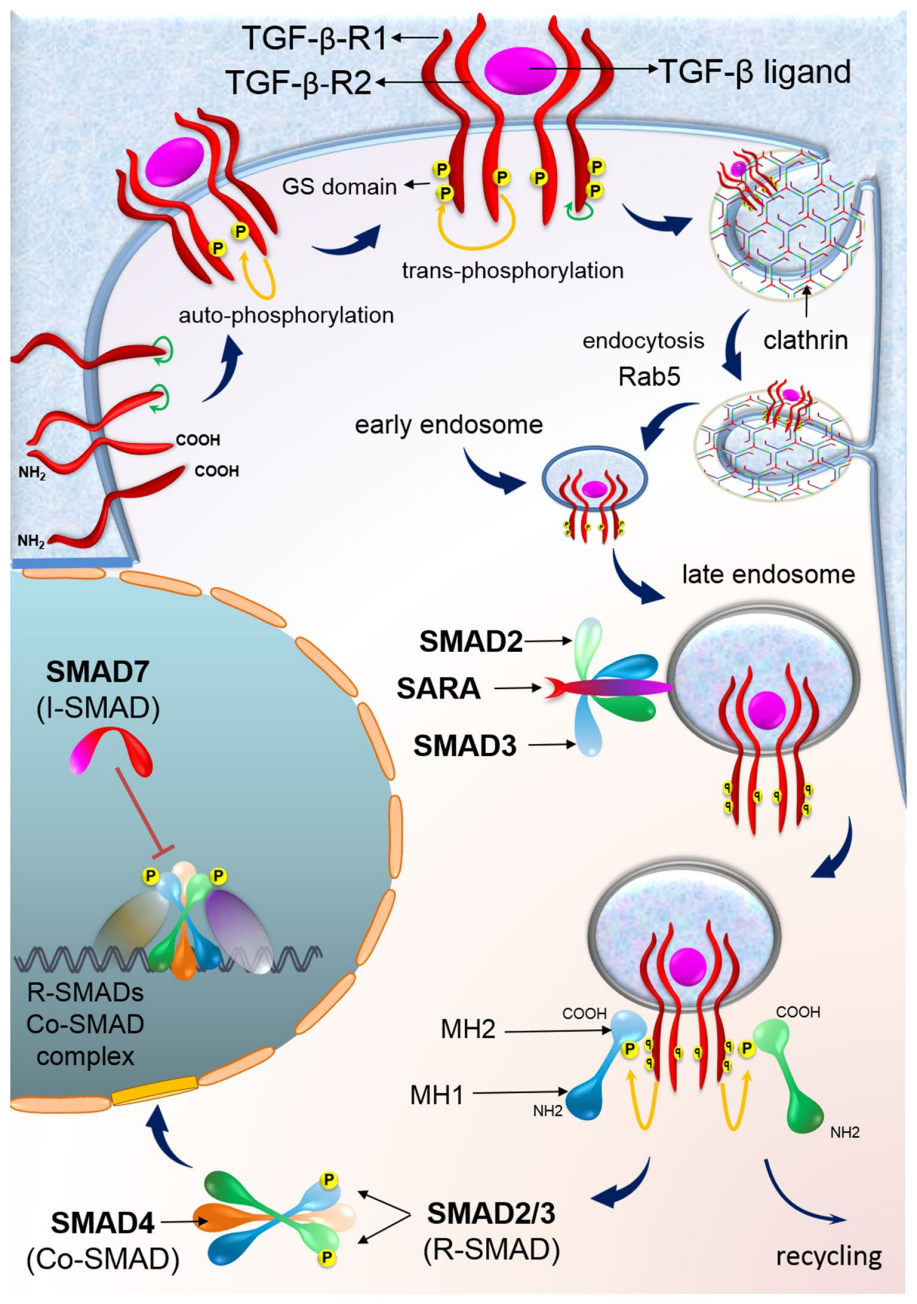
TGF-β signaling pathways in CRC. TGF-β receptors internalization occurs through clathrin-dependent or lipid-raft-dependent pathways. However, clathrin-dependent endocytosis of TGF-β receptors positively facilitates TGF-β signaling while internalization through lipid raft/caveolae exerts an inhibitory effect [70]. The internalized receptors are targeted to distinct destination through different functions of Rab5 GTPases. Binding of TGF-β ligands to TGFBR2 triggers initiation of TGF-β signaling. By binding TGF-β to TGFBR2, TGFBR2 recruits and phosphorylates TGFBR1, stimulating the protein kinase activity of TGFBR1, in which TGFBR1 is activated. Then, R-SMAD proteins or SMAD2 and SMAD3 are phosphorylated and activated by the activated TGFBR1, thereby allowing them to bind to SMAD4. As a result, the R-SMAD effectors make a complex with SMAD4 and SMAD. This complex migrates to the nucleus to regulate transcription of the target genes [28, 29]. In addition, SMAD7 has an inhibitory effect on the interaction of R-SMAD with TGFBR1 [30]. Multiple proteins contribute to the recruitment of R-SMAD proteins to the TGFBR1s and enhance SMAD activation, such as SARA [28]

TGF-β signaling pathway is involved in the control of several biological processes including cell proliferation, differentiation, migration, apoptosis and adhesion. It has been reported that TGF-β signaling reduces colon epithelial cells proliferation and promotes apoptosis and differentiation [28]. Initiation of TGF-β signaling occurs by the binding of TGF-β ligands to type II TGF-β receptors (TGFBR2). Ligand-trap proteins, which can selectively bind to specific ligands, regulate the ligand access to the receptors. By binding TGF-β to TGFBR2, TGFBR2 recruits and phosphorylates the type I TGF-β receptor (TGFBR1), thereby stimulating the protein kinase activity of TGFBR1. Then, downstream transcription factors, receptor-associated SMAD proteins (R-SMAD proteins), or SMAD2 and SMAD3 are phosphorylated and activated by the activated TGFBR1, thus allowing them to bind to SMAD4. As a result, the R-SMAD effectors make a complex with SMAD4 to translocate into the nucleus to regulate the transcription of the target genes [28, 29]. SMAD6 can inhibit the formation of R-SMAD/SMAD4 complex. In addition, SMAD7 has an inhibitory effect on the interaction of R-SMAD with TGFBR1 [30]. At the level of R-SMAD phosphorylation, other proteins including DPR2, PP2A, STRAP, EIF2A, and EIF3/TRIP1 also contribute to the inhibition of SMAD signaling. Multiple proteins contribute to the recruitment of R-SMAD proteins to TGFBR1s and enhance SMAD activation, including SARA, Endofin, Axin, DAB2 and DOK1. The ubiquitin–proteasome degradation pathway regulates steady-state SMAD protein levels (Fig. 3) [28]. Ligand binding to the TGF-β receptors cause the activation of several non-SMAD signaling and non-canonical signaling pathways. These pathways are involved in several kinase cascades of activation, such as MAPK pathways, PI3K, Notch and Wnt signaling [31]. Some important cell-cycle checkpoint genes are downstream targets of TGF-β signaling pathway such as *CDKN1A* (*p21*), *CDKN1B* (*p27*) and *CDKN2B* (*p15*). Activation of these genes causes growth arrest [32]. TGF-β inhibits normal intestinal epithelium cell proliferation and induces apoptosis and differentiation. Therefore, TGF-β acts as a tumor suppressor in the normal intestinal epithelium [33]. Many CRCs loose tumor suppressor proteins such as TGF-β in the initiation and further stages [28]. These cancers are resistant to TGF-β-induced growth inhibition [30]. However, it has been shown that TGF-β is highly expressed during the late stages of colorectal carcinogenesis, increasing the production of several mitogenic growth factors including TGF-α, FGF and EGF. As a result, it acts as a tumor promoter in the last stage of CRC [33, 34].

## Wnt signaling pathway in CRC

**Fig. 4:**
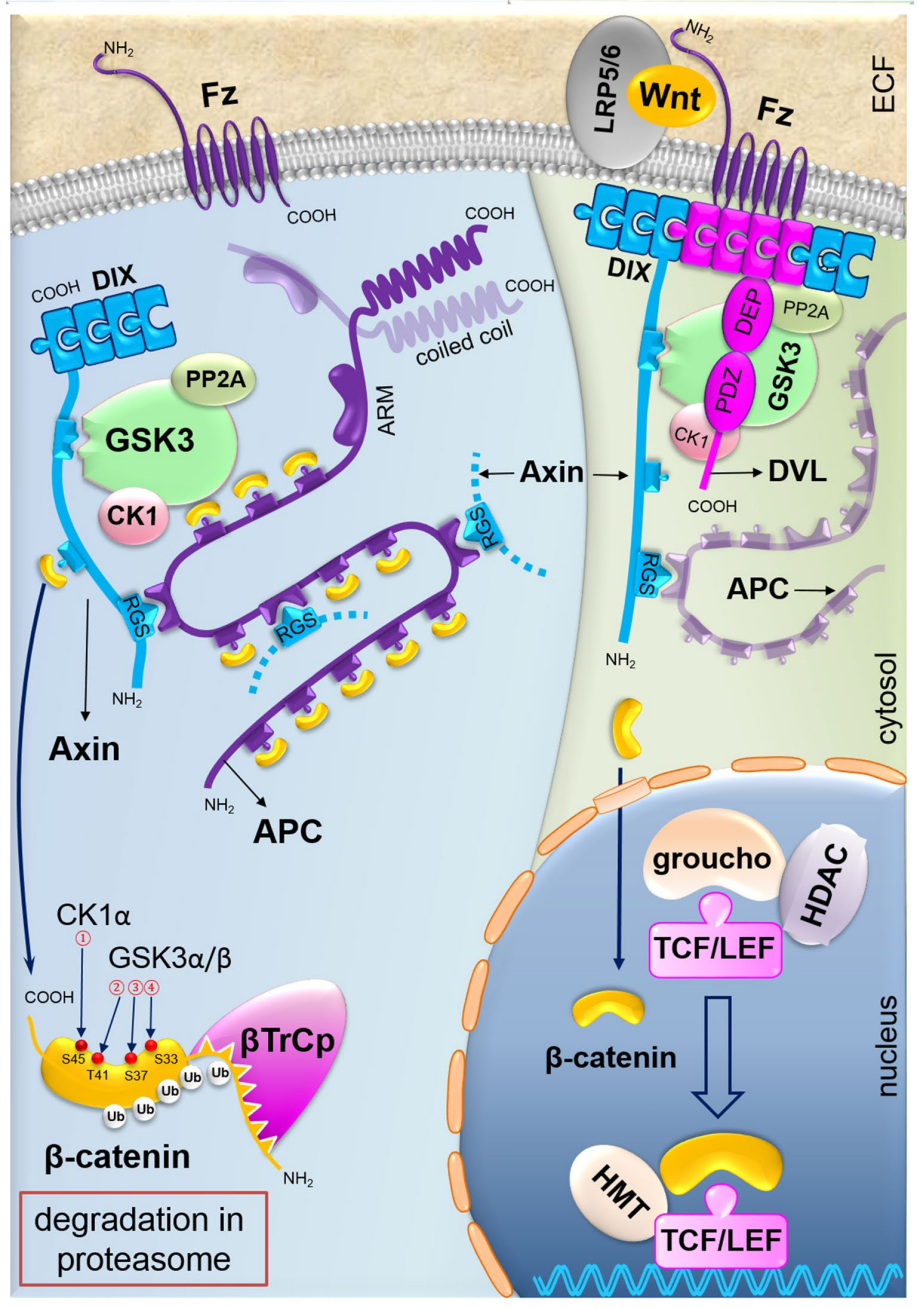
WNT signaling pathways in CRC. Accumulation of secretory Wnt ligands leads to the interaction between FZD and LRP, resulting in the activation of the DVL protein [38]. The DVL is activated and phosphorylated and translocated to the FZD receptor. The β-catenin dissociates from the degradation complex and accumulates in the cytoplasm followed by migration to the nucleus [36]. β-catenin, which is accumulated in the nucleus, could be coupled with TCF or LEF, thereby triggers activating of the expression of target genes involved in pathophysiology of CRC. These target genes are involved in the proliferation and transmission. In the absence of Wnt induction, the cytoplasmic β-catenin exists in the destruction complex, and it is phosphorylated by CK and GSK3β. Subsequently this complex recruits β-TrCP E3 linker and then degrades β-catenin via the proteasome [4]

The Wnt family consists of 19 secretory cysteine-rich glycoproteins that all play the principal regulatory roles in many developmental and biological processes such as cell fate specification, proliferation, migration and asymmetric cell division [35]. Furthermore, Wnt/β-catenin signaling serves important roles in tissue maintenance and regeneration in the hair, skin, intestine, and so on [4]. These pathways are subdivided into the canonical β-catenin-dependent and the non-canonical β-catenin-independent pathways. The canonical Wnt signaling pathway maintains crypt stem cell compartments in normal cell, thus plays complementary roles in physiology and pathology of the cell. Mutation of this pathway leads to CRC [36]. In this review, we discuss the canonical Wnt signaling pathway. The function of Wnt signaling depends on the amount of β-catenin in the cytoplasm. β-catenin could be degraded through phosphorylation and ubiquitylation [37]. These processes are performed through the destruction complex, which consists of the core proteins AXIN, adenomatous polyposis coli (APC), casein kinase 1 (CK1), and glycogen synthase kinase 3 (GSK3). When the Wnt ligands are secreted and accumulated, it leads to the interaction between frizzled (FZD) and low-density lipoprotein receptor-related protein (LRP), resulting in the activation of the DVL protein [38]. Activated type of DVL, which is phosphorylated translocated to the FZD receptor, then dissociates the β-catenin from destruction complex and further migration to nucleus [36]. β-catenin, which is accumulated in the nucleus, could be coupled with the T-cell transcription factor (TCF) or the lymphoid enhancer factor (LEF), activating the expression of target genes involved in the proliferation and transmission, such as *c*-*MYC*, *c*-*Jun*, *CCND1* (the gene encoding *cyclin D1*), *EGFR*, *CD44*, *CD133*, and leucine-rich repeat-containing receptor 5 (*LGR5*). In the absence of Wnt, the cytoplasmic β-catenin exists in the ‘destruction complex, and it is phosphorylated by CK and GSK3β; subsequently, it recruits β-TrCP E3 linker (β-transducin repeat-containing protein, an E3 ubiquitin ligase) and then degrades β-catenin via the proteasome (Fig. 4) [4]. The abnormal activation of Wnt has been observed in the human cancers, especially CRC. Published evidence shows that hyperactivation of Wnt plays an oncogenic role in CRC [39]. Activation of Wnt signaling pathway is required for tumor growth in advanced CRC, therefore this pathway can be an effective therapeutic target for CRC treatment [36].

## Mutations in the components of signaling pathways

The mutations in some of the tumor suppressors or oncogenes have been implicated in CRC development. Here, we summarize a number of mutations in the genes involved in these signaling pathways in CRC. It has been reported that EGFR modulations occur in CRC, such as EGFR overexpression, EGFR mutations, gene amplification, and copy number [9]. K-Ras proto-oncogene mutation is one of the early events in the development of this cancer. Approximately, 40% of CRCs have K-Ras mutations [40]. In contrast, N-Ras mutations have been observed in about 2.5% to 4.5% of CRCs [41]. Mutations of B-Raf have been observed frequently in the sporadic CRC with microsatellite instability (MSI). Approximately, 8% of CRCs have point mutations in B-Raf. Mutations of Ras exist more in B-RafD594G mutant CRCs, rather than B-RafV600E mutant CRCs. The B-RafV600E mutation is associated with poor prognosis [42]. Among the mechanisms causing the increase in ERK–MAPK pathway in CRC, upregulation of the EGFR is a potential mechanism contributing to MAPK activation in this cancer [43]. The tumor initiation mechanisms of Notch signaling in CRC are not well understood. Overexpression of mutated Notch receptors, ligands, and Notch signaling interplays between signaling pathways [20]. However, similar to other cancers, molecular genetic alterations in the loci of the Notch receptor (such as chromosomal translocation, point mutations, and chromosomal amplification) may play several roles [44]. Overall, no significant mutations have been reported in Notch signaling components [45]. Nevertheless, Malapelle et al. reported that one case which had mutation in Notch-1, while this case carried other mutations in TP53, KRAS, PTEN, ERBB4 and PIK3CA genes [46]. Studies have also shown that alterations in PI3K pathway occur in CRC. The activation of this pathway, either through mutations in PIK3CA and PIK3CB, or amplification of AKT, loss of the function of PTEN and the hyper-activation of mTORC1, could contribute to the malignancy of the benign lesions [25]. PIK3CA gene mutations occur in approximately 10–20% of CRCs [47]. In 20–40% of CRCs, mutations in the PTEN gene have been observed [48]. It has been reported that some of the mechanisms by which tumors can escape the TGF-β-induced growth inhibition are mutations in the receptors, R-SMAD proteins, or SMAD4. Overexpression of inhibitory SMAD6 or SMAD7 proteins, blocking phosphorylation of R-SMAD proteins, or increased ubiquitin-mediated proteolysis should also be noted [28, 49]. Approximately, 20–30% of CRCs contain *TGFBR2* mutations, especially frameshift mutations, are mostly detected in colon cancer cells with MSI [49]. Studies have shown that one of the factors contributing to the transformation of CRCs is the inactivation of *TGFBR2* [28]. As in the absence of *TGFBR2*, cellular proliferation is increased due to the prolonged activation of cdk4 expression [34]. Identified mutations in TGFBR1 in CRC cell lines are not common [50]. TGFBR1*6A, a polymorphic allele of *TGFBR1*, was detected in the *TGFBR1* tumor-specific mutations [51]. TGFBR1*6A has been associated with a 24% increase in the risk of CRC [28, 51]. It is speculated that TGFBR1*6A may cause switching of TGF-β growth inhibitory signals to the growth stimulatory ones, thereby contributing to tumorigenesis [51]. SMAD2 mutations occur in approximately 3–6% of CRC tumor cases [52]. However, SMAD3 mutations are infrequent in tumors [50]. Disruption of *SMAD4* is most commonly found in cancers. *SMAD4* mutations have been identified in 20–30% of CRCs, but mutations in other *SMAD* genes have been less identified. The results of previous studies show that loss of *SMAD4* expression contributes to colorectal carcinogenesis [28]. One of the main genomic alterations associated with TGF-β pathway inactivation is loss of chromosomal 18q. Since the two important tumor suppressor genes, SMAD2 and SMAD4 are located on this part of chromosome, the loss of chromosome 18q inactivates this pathway and promotes the evasion of apoptosis and cell proliferation [53]. Of note that in CRCs, frequently mutations occur in two genes: APC and CTNNB1 [4]. Mutations in APC leads to the activation of Wnt pathway, a critical event in colorectal tumorigenesis. APC mutations are observed in approximately 80% of CRCs. Approximately 5–10% of mutations exist in other components of Wnt signaling, such as the CTNNB1 gene [40]. Mutations of the β-catenin gene are not coincided with the APC mutations as half of all CRCs with mutations in the CTNNB1 gene have the wild-type APC gene [54]. It has also been reported that the frequency of AXIN mutations in CRC is around 6% (Table 1) [55].

**Table 1:** Components of the signaling pathway involved in CRC and their abnormalities

(Gene/Protein)	Observed abnormalities in CRC	Frequency in CRC	References
*EGFR*/EGFR	Protein expression Mutation Increased copy number Increased expression	25–90% Rare 0–50% 30–90%	[9] [14] [5]
*K*-*ras/*K-Ras	Activating mutation	30–40%	[40]
*N*-*ras/*N-Ras	Activating mutation	2.5–4.5%	[41]
*B*-*Raf/*BRAF	Activating mutation (V600E)	8%	[42]
*PIK3CA/*PI3KCA	Activating mutation (exons 9 and 20)	10–20%	[46]
*PTEN/*PTEN	Loss of protein expression Mutation Loss of heterozygosity	20–40%	[69]
Components of NOTCH	Increased expression	–	[21]
*TGFBR2*/TGFBR2	Mutation	20–30%	[48]
*TGFBR1/*TGFBR1	TGFBR1*6A	24%	[27]
*SMAD2/*SMAD2	Mutation	3–6%	[68]
*SMAD4/*SMAD4	Mutation	20–30%	[27]
*SMAD3/SMAD3*	Mutation	Infrequent	[49]
*APC*/APC	Mutation	80%	[40]
*CTNNB1/*CTNNB1	Mutation	5–10%	[40]
*AXIN/*AXIN	Mutation	6%	[55]

## Crosstalk of signaling pathways

**Fig. 5:**
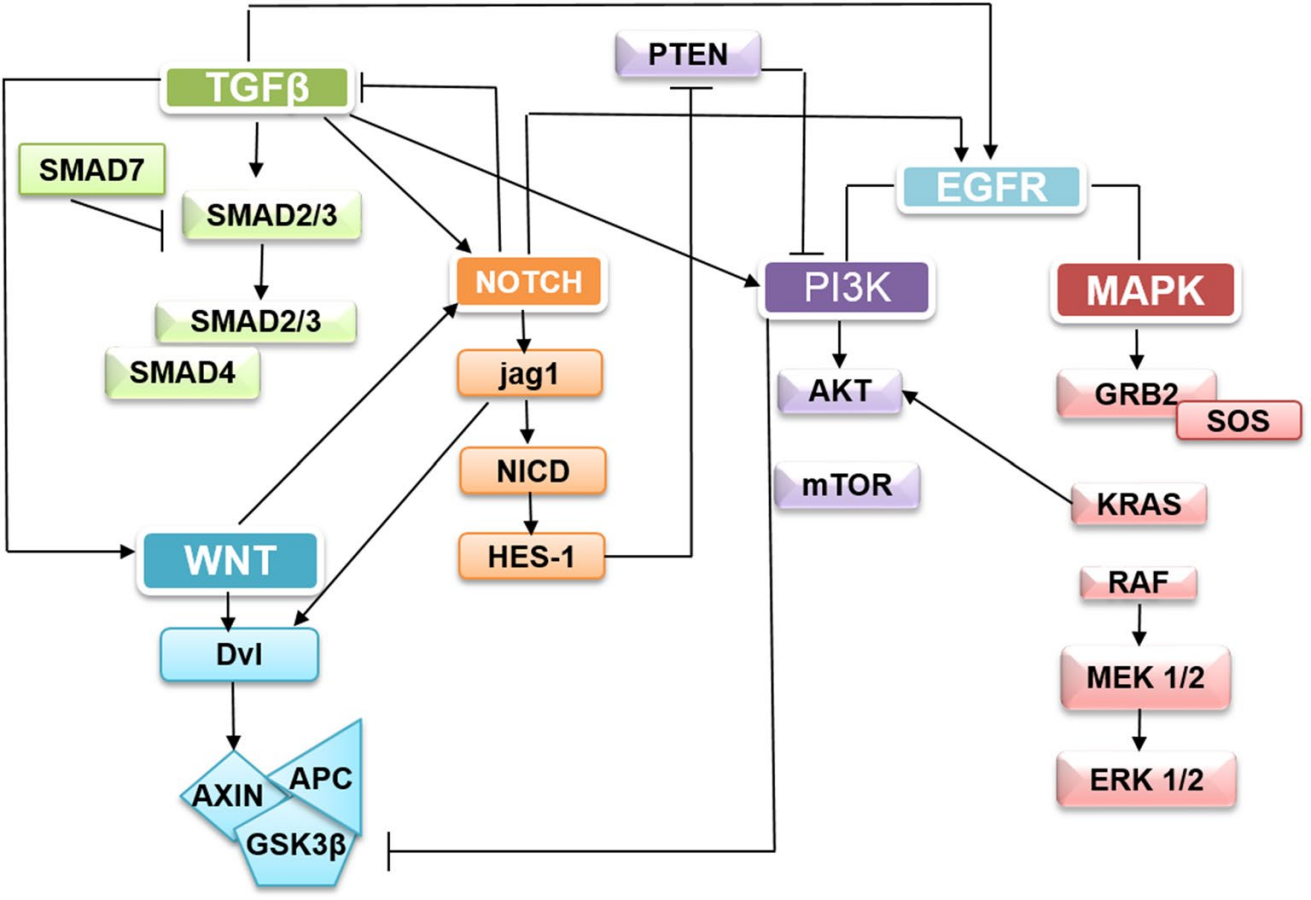
The Notch, PI3K, TGF-β, Wnt/B-catenin, and EGFR/Ras signaling pathways are interacting with each other. Notch signaling regulates Wnt pathway through disheveled and Wnt signaling actives Jagged-1 [56]. Also Notch stimulates EGFR and suppresses the activity of TGF-β pathway [20, 57]. HES1 inhibits PTEN thus activates PI3/AKT pathway in CRC [20]. PI3K can inactivates GSK3β as their downstream target and KRAS can actives PI3K [58]. TGF-β superfamily signaling can activates oncogenic pathways (PI3K/AKT, MAPK/ERK, WNT, and Notch) in CRC [31]

The first report on the crosstalk between Wnt and Notch signaling was reported in fruit fly which showed NICD can bind to disheveled one and the subsequent Notch signaling and regulate the Wnt pathway. Wnt/β-catenin signaling increases the Notch expression and triggers onset of CRC through activation of Jagged-1 [56]. Notch stimulates EGFR pathway during colonic carcinogenesis. It is reported that HES1 inhibits PTEN thus Notch signaling activates PI3/AKT pathway in CRC through activation of HES1 [20]. In addition, Notch signaling suppresses the activity TGF-β pathway which is important for cell growth inhibition. Induction of Jagged-1 by TGF-β, leads to Notch overexpression in CRC [57]. TGF-β superfamily signaling independent of SMADs (non-canonical pathways) can activate a number of CRC oncogenic pathways, including but not limited to PI3K/AKT, MAPK/ERK, Wnt, and Notch [31]. PI3K plays an actual role in the context of tumorigenesis through regulating β-catenin signaling. It has been suggested that PI3K can inactivate GSK3β as a downstream target [58]. KRAS can interact directly with the PI3K catalytic subunit and then activate it. It has also been reported that the interaction between the APC–Axin–GSKb3 complex and SMAD7 leads to modulation in the activity of SMADs [30] (Fig. 5). These dysregulations in the signaling pathways contribute to unchecked cell cycle progression, the increase of cell proliferation, the escape of apoptosis, and enhanced invasiveness and metastasis [59]. The expression and interaction of G1 cyclins) cyclins D and E(, CDKs (CDK4/6 and CDK2), and CDK inhibitors including p16 family (p15, p16, p18 and p19) and p21 family (p21, p27, p28 and p57) regulate the cell cycle. These cell cycle regulators are dysregulated in CRC [5]. It has been reported that overexpression of cyclin D1 is related to the aberrant accumulation of β-catenin as a result of APC mutation and oncogenic K-Ras activation. Both events act in a cooperative manner to regulate the cyclin D1 expression [4]. Activation of PI3K–Akt signaling leads to the induction of the coordinated expression of cyclin D1, cyclin E and CDK4 [60]. A study has shown that MAML-1, Notch pathway co-activator, can bind to the promoters of two genes related to the cell cycle progression, cyclin D1 and c-Myc in the colon cancer cell lines. Therefore, the anticancer effect of Notch inhibition is related to its inhibitory effect on the cell cycle progression [45]. Several members of the p16 and p21 families are downregulated in the CRC tissues. It should be noted that methylation of p16 gene is associated with K-Ras mutation. p21, is a p53 target gene and TGF-β signaling enhances the protein stability of p53 [5]. It has been observed that PI3K–Akt inhibition leads to the upregulation of expression of p21 and p27 [27]. Apoptosis is mostly dysregulated in the human cancers such as CRC. In CRC, pro-survival signaling pathways inhibition such as PI3K may also enhance death receptor-mediated apoptosis [5]. Activation of the Notch signaling could upregulate the expression of apoptosis inhibiting genes including Bcl-2, Bcl-XL, XIAP and Survivin [20, 61, 62]. The Notch signaling has been reported to reduce the expression of KLF4, an essential factor for inhibiting cell proliferation and maintenance of the terminal differentiation of goblet cells [63]. Also, β-catenin induces Bcl-2 expression through its downstream targets. The expression of Survivin and XIAPs has been shown to be positively regulated by PI3K–Akt, Notch, Ras, Wnt/β-catenin and negatively by TGF-β [5].

## Strategies for the therapy targeting components of signaling pathways in CRC

Disruptions of these pathways are closely associated with the occurrence and development of CRC. These signaling cascades activation leads to malignant phenotypes acquisition. However, their relative importance and interim relevance in colorectal carcinogenesis are not still completely understood [40]. One novel molecular therapeutic approach is to target these pathways with specific inhibitors. Small molecule inhibitors provide excellent opportunities for researchers to study the multiple aspects of cell biology, ranging from cell cycle control and mitosis to signaling pathways and gene expression. In general, studies have proposed four compounds to target PI3K signaling. These compounds include: PI3K inhibitors, dual inhibitors of PI3K and mTOR, AKT inhibitors and mTOR inhibitors [25]. Recently, Wnt inhibitors are developed and have reached to a promising start, whereas an approved clinically effective drug is still missing in CRC. It have been reported that various types of therapeutic agents modulating Wnt pathway are ranging from small-molecule compounds to antibodies and peptides. Wnt inhibitors have been summarized into four classes based on their specific targets: generic, the Wnt-receptor complex, the β-catenin destruction complex and nuclear/transcription factor complexes [36]. Effective method of therapeutic strategies against TGF-β are classified into the ligand, ligand-receptor and intracellular levels. Development and testing of powerful anti-TGF-β strategies are carried out in pre-clinical studies as well as clinical trials [64]. In CRC, antisense molecules by binding to specific mRNA, can prevent TGF-β synthesis on the ligand level. Also, it is mentioned that the kinase activity of TGF-β R1 and TGF-β R2 can be inhibited by a small molecule (LY2109761) [59].

Various in vitro and in vivo studies have shown the knockdown of Notch receptors and Notch ligands by the small interfering RNA (siRNA) with various therapeutic effects [20, 45]. Targeting Notch signaling has also been obtained using antagonizing antibodies against Notch receptors or ligands. On the other hand, chemical inhibitors of γ-secretase are used in Notch signaling targeting [20, 59]. It has also been reported that EGFR, Ras, Raf and Mek inhibitors, and EGFR antibodies are interfering in MAPK signaling targeting at cytosol (Table 2) [14]. Colorectal cancer cells directly could be affected by EGFR-targeting drugs as accumulating drug resistance mutations in the RAS–RAF–MEK–ERK pathway affects these cells [65]. The main agent available for RAS wild-type (WT) metastatic colorectal cancer (mCRC) treatment is anti-EGFR mAb such as cetuximab or panitumumab [66]. Moreover, panitumumab is a fully human mAb with the properties similar to cetuximab [67]. Combination of targeting multiple signaling pathways with conventional chemotherapeutics may also enhance the treatment efficacy [5]. Therefore, the priority of these FDA approved drugs and the decision of treatment option between them depend mostly on the patient condition and clinical availability which should be considered by physicians.

**Table 2:** Small molecule inhibitors of the signaling pathways and their target genes (5)

Signaling pathways	Factors/drugs approved (or) in development	Function	Target
EGFR–RAS–MAPK	Cetuximab	EGFR inhibitor	EGFR
	Panitumumab	EGFR inhibitor	EGFR
	Gefitinib	EGFR inhibitor	EGFR
	Sorafenib	Tyrosine kinase inhibitor	RAF
	Pertuzumab	Monoclonal antibody for Her 2	Her2
	Erlotinib	Receptor tyrosine kinase inhibitor	EGFR
	Canertinib (CI-1033)	Irreversible tyrosine kinase inhibitor	EGFR Her2 ErbB-4
	Lapatinib	Tyrosine kinase inhibitor	EGFR Her2
	Dacomitinib	Irreversible EGFR inhibitor	EGFR
	Sunitinib	Receptor tyrosine kinase inhibitor	RTKs
	PD158780	Tyrosine kinase inhibitor	EGFR ErbB-3 ErbB-4
	Matuzumab (EMD72000)	Monoclonal antibody for EGFR	EGFR
	Vemurafenib (PLX4032)	BRAF^V600E^ inhibitor	BRAF^V600E^
	Dabrafenib (GSK2118436)	BRAF^V600E^ inhibitor	BRAF^V600E^ C-RAF BRAF
	Encorafenib (LGX818)	RAF inhibitor	BRAF^V600E^
	XL281(BMS-908662)	RAF kinases inhibitor	RAF
	Trametinib (GSK1120212)	MEK1/2 inhibitor	MEK1/2
	Binimetinib	MEK1/2 inhibitor	MEK1/2
	Selumetinib (AZD6244)	MEK inhibitor	MEK1/2
	VX-11e	ERK2 inhibitor	ERK2
	SCH772984	ERK1/2 inhibitor	ERK1/2
NOTCH	RO4929097	γ Secretase inhibitor	γ Secretase
	DAPT (GSI-IX)	γ Secretase inhibitor	γ Secretase
	Dibenzazepine (DBZ)	γ Secretase inhibitor	γ Secretase
	LY411575	γ Secretase inhibitor	γ Secretase
	GSI34	γ Secretase inhibitor	γ Secretase
PI3K–Akt–mTOR	OSI027	mTOR kinase inhibitor	mTOR PI3Kγ
	MK2206	AKT inhibitor	AKT
	GSK690693	AKT inhibitor	AKT
	Everolimus (RAD001)	mTOR inhibitor	mTOR
	Idelalisib (CAL-101)	P110 inhibitor	P110γ/δ
	BGT226	PI3K/mTOR inhibitor	mTOR PI3Kα/β/γ
	Buparlisib (BKM120)	PI3K inhibitor	P110α/β/γ
	Dactolisib (NVP-BEZ235)	PI3K and mTOR inhibitor	mTOR P110α/γδ
	GSK690693	Akt inhibitor	AKT
	AZD8055	mTOR inhibitor	mTOR
	Alpelisib (BYL719)	PI3Kα inhibitor	PI3Kα
	Perifosine	Akt inhibitor	AKT
	SF-1126	Inhibitors of the PI3K pathway	PI3K mTOR
	Pilaralisib (XL147)	PI3K inhibitor	PI3Kα/β/γ/δ
	Triciribine (VQD002)	Akt inhibitor	AKT
	Temsirolimus	Kinase Inhibitor	mTOR
TGF-β	LY2109761	TGF-β receptor inhibitor	TGFBRI/II
	AP15012	Antisense molecules	TGF-β1
	AP11014	Antisense molecules	TGF-β
Wnt/β-catenin	BC2059	β-catenin inhibitor	β-catenin
	Sulindac	Blocking the PDZ domain of the protein Dishevelled	Dishevelled
	PNU-74654	Wnt/B-catenin inhibitor	Wnt/B-catenin

## Conclusion

In this review, we attempted to summarize the molecules, mechanisms, cross-talks and the corresponding targeted therapies involved in colorectal progression. In the future, it is also expected that some of mentioned inhibitors would come to the FDA’s approval phase and could be used to treat the patients with CRC. It is suggested to perform more research in the field of SMAD inhibitors in the future, as SMAD proteins are the downstream of TGF-β signaling. Finding of potent SMAD inhibitors may help to inhibit several non-canonical pathways activated by TGF-β. On the other hand, it has been shown that accumulation of different mutations can contribute to tumor heterogeneity, causing a unique biologic phenotype formation for each cancer individual. Therefore, it is recommended to identify and evaluate these mutations. Despite extensive studies on the role of signaling pathways in the development of CRC, we need to improve our knowledge of the interaction between the components of these pathways to find new therapies for CRC.

## Data Availability

Not applicable.

## Abbreviations

APC: adenomatous polyposis coli
Co-Smads: common partner Smads
CRC: colorectal cancer
DLL1: delta-like protein 1
Dvl: dishevelled
EGF: epidermal growth factor
Fz: frizzled receptor
Grb2: growth factor receptor-bound protein 2
GS domain: glycine-serine repeats domain
Hey1: hairy/enhancer-of-split related with YRPW motif protein 1
Hes1: Hes family of bHLH transcription factor 1 that bind to the enhancer box (E-box)
I-Smads: inhibitory Smads
JAG1: Jagged-1
LNR: Lm-Notch receptors
MH1/2 domain: Mad-homology1/2 domain
NLS: nuclear localization signal
NECD/NEXT: Notch extracellular truncation product
PP2A: protein phosphatase 2A
RBPJ: recombining binding protein suppressor of hairless for immunoglobulin kappa (Igκ) region
R-Smads: receptor-regulated Smads
RTK: receptor tyrosine kinase
SARA: SMAD anchor for receptor activation
siRNA: the Small interfering RNA
TGF-β/TGFB: transforming growth factor beta
TGF-βR1/TGFBR1: transforming growth factor beta receptor 1
TMD/TMR: transmembrane domain/region
TSC1/2: tuberous sclerosis 1 or 2
Ub/Ubq: ubiquitin
ZFYVE9: zinc finger FYVE domain-containing protein 9
